# Sex-specific chrono-nutritional patterns and association with body weight in a general population in Spain (GCAT study)

**DOI:** 10.1186/s12966-024-01639-x

**Published:** 2024-09-12

**Authors:** Luciana Pons-Muzzo, Rafael de Cid, Mireia Obón-Santacana, Kurt Straif, Kyriaki Papantoniou, Isabel Santonja, Manolis Kogevinas, Anna Palomar-Cros, Camille Lassale

**Affiliations:** 1 ISGlobal, Barcelona, Spain; 2 Universitat Pompeu Fabra (UPF), Barcelona, Spain; 3 Genomes for Life -GCAT lab Group, Program of Predictive and Personalized Medicine of Cancer (PMPPC), Institute for Health Science Research Germans Trias i Pujol (IGTP), Badalona, Spain; 4 Oncology Data Analytics Program (ODAP), Unit of Biomarkers and Suceptibility (UBS), Catalan Institute of Oncology (ICO), L’Hospitalet del Llobregat, Barcelona, Spain; 5 ONCOBELL Program, Bellvitge Biomedical Research Institute (IDIBELL), L’Hospitalet de Llobregat, Barcelona, Spain; 6 Consortium for Biomedical Research in Epidemiology and Public Health (CIBERESP), Instituto de Salud Carlos III (ISCIII), Madrid, Spain; 7 Boston College, Boston, MA USA; 8 Department of Epidemiology, Center for Public Health, Medical University of Vienna, Vienna, Austria; 9 Department of Social and Preventive Medicine, Center for Public Health, Medical University of Vienna, Vienna, Austria; 10 IMIM (Hospital del Mar Medical Research Institute), Barcelona, Spain; 11 Consortium for Biomedical Research - Physiopathology of Obesity and Nutrition (CIBEROBN), Instituto de Salud Carlos III (ISCIII), Madrid, Spain

**Keywords:** Chrono-nutrition, Meal timing, Circadian rhythm, Obesity, Body mass index

## Abstract

**Background:**

Altered meal timing patterns can disrupt the circadian system and affect metabolism. Our aim was to describe sex-specific chrono-nutritional patterns, assess their association with body mass index (BMI) and investigate the role of sleep in this relationship.

**Methods:**

We used the 2018 questionnaire data from the population-based Genomes for Life (GCAT) (*n* = 7074) cohort of adults aged 40–65 in Catalonia, Spain, for cross-sectional analysis and its follow-up questionnaire data in 2023 (*n* = 3128) for longitudinal analysis. We conducted multivariate linear regressions to explore the association between mutually adjusted meal-timing variables (time of first meal, number of eating occasions, nighttime fasting duration) and BMI, accounting for sleep duration and quality, and additional relevant confounders including adherence to a Mediterranean diet. Finally, cluster analysis was performed to identify chrono-nutritional patterns, separately for men and women, and sociodemographic and lifestyle characteristics were compared across clusters and analyzed for associations with BMI.

**Results:**

In the cross-sectional analysis, a later time of first meal (β 1 h increase = 0.32, 95% CI 0.18, 0.47) and more eating occasions (only in women, β 1 more eating occasion = 0.25, 95% CI 0.00, 0.51) were associated with a higher BMI, while longer nighttime fasting duration with a lower BMI (β 1 h increase=-0.27, 95% CI -0.41, -0.13). These associations were particularly evident in premenopausal women. Longitudinal analyses corroborated the associations with time of first meal and nighttime fasting duration, particularly in men. Finally, we obtained 3 sex-specific clusters, that mostly differed in number of eating occasions and time of first meal. Clusters defined by a late first meal displayed lower education and higher unemployment in men, as well as higher BMI for both sexes. A clear “breakfast skipping” pattern was identified only in the smallest cluster in men.

**Conclusions:**

In a population-based cohort of adults in Catalonia, we found that a later time of first meal was associated with higher BMI, while longer nighttime fasting duration associated with a lower BMI, both in cross-sectional and longitudinal analyses.

**Supplementary Information:**

The online version contains supplementary material available at 10.1186/s12966-024-01639-x.

## Background

Prevalence of obesity has dramatically increased over the last decades in all world regions [1] with an estimated 1.9 billion adults having excess weight (body mass index [BMI] ≥ 25 kg/m^2^), of which 650 million have obesity (BMI ≥ 30 kg/m^2^) [2]. Obesity is a major public health problem, as it is a condition with multifactorial origins and many health ramifications [3]. Important modifiable risk factors for obesity include unhealthy eating behaviors, not getting enough good-quality sleep, and lack of physical activity [4]. Regarding eating behaviors, a growing field of research known as “chrono-nutrition” focuses on the health impacts of *when* we eat besides *what* we eat.

The circadian system regulates the sleep-wake and feeding-fasting cycles, organizing a sequence of physiological processes throughout the day over approximately 24 h [5], synchronized every day to external time givers via the suprachiasmatic nuclei in the hypothalamus, which is sensitive to external light via the retina [6]. Food intake is another important external cue for peripheral circadian clocks. Communication between the central and peripheral circadian clocks occurs through neural and hormonal pathways, ensuring the synchronization of bodily functions and behaviors with the daily light-dark cycle [6, 7]. Furthermore, the central clock processes light cues and synchronizes the peripheral clocks [8]. Unusual eating patterns (e.g. late-night eating) can disrupt the circadian system through decoupling of peripheral oscillators [9]. The relationship between sleep and meal timing is intricate. Misalignment between circadian rhythms and behaviors like eating and sleeping can disrupt metabolic processes governed by energy metabolism and appetite-regulating hormones. This misalignment can lead to adverse metabolic consequences and an increased risk of obesity when these behaviors consistently fail to synchronize with circadian cues [10, 11]. Various peripheral clocks in different organs such as the gut, muscles, adipose tissue, liver, and pancreas are responsible for regulating insulin secretion and adiposity [9, 12]. Moreover, the circadian system is also affected by hormonal changes. An important life stage of hormonal changes in adult women is menopause, that also includes sleep disorders and circadian modifications [13].

Data suggest that breakfast consumption plays an important role in the maintenance of homeostatic control, with breakfast skipping being associated with metabolic and inflammatory deregulation [14–16]. Epidemiological studies have reported that breakfast skipping relates with increased risk of overweight, obesity, an adverse metabolic profile, and cardiovascular diseases, independent of diet quality, when compared to breakfast eaters [17]. Behavioural data have shown that eating breakfast reduced overall fat intake and minimized compulsive snacking [17] and skipping breakfast was associated with an unhealthy diet and higher energy intake during the day [18]. On the other hand, food intake close to sleep time may be associated with poor sleep quality and nocturnal awakening [19, 20]. Late sleep times and late rise times were found to be associated with later eating times and increased caloric intake in a cross-sectional study [21], and eating less than 3 h before sleep time was associated with 40% greater odds of presenting nocturnal awakening [19]. Sleep restriction also increased intake of calories from snacks and overall energy intake in adults [22]. Intervention studies have shown that eating an early dinner could help achieve a reduction in body weight and fat [23, 24]. Moreover, whilst short-term intervention trials (~ 8 weeks) suggest that skipping breakfast can help lose weight [25], long-term studies have shown that consuming breakfast and eating the largest meal in the morning, combined with an appropriate length of overnight fasting, may be effective for preventing long-term weight gain [26]. Despite this emerging evidence, there is a lack of descriptive evidence of chrono-nutritional patterns in Spanish population and their relationship with adiposity.

Cluster analysis can help advancing our understanding of chrono-nutrition and its relationship with health outcomes by allowing a holistic examination of complex interactions among nutritional and lifestyle aspects, grouping individuals based on similar behaviors. A study performed in Austria [27], the first one using this approach, found that participants in a cluster reporting longer fasting intervals and higher frequency of breakfast skipping, had a higher prevalence of chronic insomnia, depression, obesity, and poor self-rated health status. However, traditional eating patterns in Spain include later meal timings in comparison to other European countries [28], especially northern Europe, where meal timings are more similar to the ones in the United States [29]. The Spanish national dietary guidelines do not have recommendations regarding meal timing, but some reports [30, 31]recommend that breakfast should contribute 20–25% of the total daily energy intake with adequate variety of food groups.

The cluster approach becomes particularly relevant when considering sex differences in nutritional patterns, a phenomenon noted in studies across diverse cultural settings that often found women exhibiting healthier eating habits than men [32–34]. Incorporating an understanding of gender roles and social aspects into sex-specific analyses is crucial. However, to the best of our knowledge, no study has previously examined chrono-nutritional patterns considering sex-specific differences.

Our main objective was to describe chrono-nutritional patterns and assess their relationship with body mass index (BMI), and to explore the role of sleep (quality and quantity) as a potential mediator in this association. Moreover, sex differences in socioeconomic and lifestyle factors might impose differences between men and women in chrono-nutritional patterns, therefore we investigated these relationships separately for men and women.

## Methods

### Study design and population

The Genomes for Life (GCAT) study [35] is a population-based prospective cohort study, designed to study the role of environmental, and genetic factors in the development of chronic diseases in the adult population of Catalonia. The study recruitment was open to any volunteer that was interested in participating; however, most of the cohort participants were invited through the Blood and Tissue Bank (BST), a public agency of the Catalan Department of Health. Participants were invited to participate using multiple strategies (phone calls, mail, GCAT website or in person). Inclusion criteria to participate in the study include age 40–65 years old, understand at least one of the two official languages, provide written consent, possess an individual Health System Identification Card, and be current residents of Catalonia. Exclusion criteria includes mental or health impairment disorders to give written informed consent or efficient communication or planning to leave Catalonia in the next five years. The baseline visit was in 2014–2017 and a first follow-up (Follow-up 1) questionnaire was filled out in 2018. The GCAT cohort study was approved by a local Ethics committee and informed consent of participants was obtained. The data from the GCAT cohort was extracted in an anonymized format, ensuring participants could not be identified.

We only included in this study participants who responded to Follow-up 1 questionnaire in 2018, at which the detailed diet assessment through a validated semiquantitative food-frequency questionnaire (FFQ) was performed (*n* = 9270). The FFQ [36], containing the food groups most eaten in Spain, is a Spanish validated full-length questionnaire with more than 128 questions, with a time frame referring to the previous 12 months. The validation study used four sets of 3-day dietary records over one year as reference to explore validity, in participants aged 55–80 years old; in this study, reproducibility for food groups, energy and nutrient intake, explored by the Pearson correlation coefficient ranged 0·50–0·82. Subjects with missing data on our main exposures (*n* = 327) and covariates (*n* = 1156) were also excluded. In particular, there were missing data from the FFQ. Further, shift workers were excluded, as this type of work can alter circadian rhythms (eating habits, physical activity, sleep) and lead to chrono-disruption (*n* = 661) [37]. This resulted in an analytical sample of 7074 subjects (Figure S1). Baseline population (*n* = 19,392) is briefly described and compared with our analysis sample, which is younger and have a greater percentage of higher education, in Table S1.

The main analyses are cross-sectional using data from the Follow-up 1 questionnaire. We used data from a second follow-up questionnaire (Follow-up 2) sent out between January and May 2023 [38] to perform a longitudinal analysis, although this was on a subsample only (*n* = 3128).

### Data collection and variables assessment

In the baseline visit, anthropometric and other clinical measurements were taken, and participants answered a computer-based questionnaire. Anthropometric measurements were taken by trained personnel using a defined protocol on the baseline visit [35]. Information on demographic (age, sex), socioeconomic data (education, employment), and measured height obtained from the baseline visit were used for the analysis. The Follow-up 1 questionnaire assessed body weight, and included questions regarding personal habits (alcohol consumption, smoking, physical activity), the Mental Health Inventory score (MHI5) [39], night shift work history and an FFQ, from which a Mediterranean diet adherence score, the rMED [40] was calculated. The rMED includes 9 components, which are calculated as a function of energy density (g · 1000 kcal − 1 · d − 1). A score of 0 to 2 was assigned to the first, second, and third tertiles of intake for the 6 beneficial components: fruit & nuts, vegetables (excluding potatoes), legumes, fish, and cereals, olive oil and the scoring was inverted for the 2 detrimental components: meat and dairy products. Regarding alcohol, a score of 2 was assigned for moderate consumers (range: 5–25 g/d for women and 10–50 g/d for men) and of 0 for subjects outside (above or below) the sex-specific range. The total score ranges from 0 to 18 with higher scores reflecting greater adherence to the Mediterranean diet. Adherence levels were classified into: low (0–6), medium (7–10), and high (11–18). Besides, an entire section of that questionnaire was dedicated to circadian behaviours: meals and snacks consumption and timing, sleep habits, self-reported sleep satisfaction and chronotype. Chronotype was self-reported. The question had multiple choice options for answer: clearly morning type, more morning than evening, more evening than morning, clearly evening. Sleep dissatisfaction was self-reported in one question “Are you satisfied with your sleep?”, with possible answers “Never”, “sometimes” and “often”, and coded as no (“often”) and yes (“never”, “sometimes”).

#### Exposure assessment

Usual consumption (yes/no) and average timing of the 3 main meals of the day (breakfast, lunch, and dinner) and 2 snacking times (afternoon and late-night) were self-reported in a 24-hour format, separately for weekdays and weekends, on average over the last 12 months. The number of eating occasions was then derived as a discrete variable (ranging 1 to 5). The variables “time of first meal” and “time of last meal” were calculated using the time of first and last eating occasion, respectively. Time of first meal was separated into a dichotomous variable (before/after 8:30 h) for effect modification exploration. This cut-off choice was data-driven (mean time of first meal in the overall population) and has been used in previous studies [41]. Nighttime fasting duration was calculated as the time in hours between the last meal of a day and the first meal of the following day as was the time between the last meal and sleep. Average sleep time, on weekdays and weekends separately was also self-reported in the follow-up questionnaire and used to calculate the time between last meal and sleep. For more details regarding the questionnaire, see Supplemental Text. The eating midpoint [42] was calculated as follows to evaluate the variability of meal timing during weekdays and weekends:

Eating midpoint (local time) = [(Time of the last meal – Time of the first meal)/2] + Time of the first meal.

#### Outcome assessment

Height from the baseline visit (2014–2017) and self-reported weight from the first follow-up (2018) and second follow-up (2023) were used to calculate BMI as weight in kilograms divided by squared height in meters. For all timing variables, the beta coefficients represent the BMI difference in kg/m^2^ for one-hour increase in the exposure.

### Statistical analysis

Normality was inspected using histograms, skewness and kurtosis. Descriptive statistics were performed to describe participants’ characteristics, using means and standard deviations for continuous variables, except for non-normal variables, for which median and interquartile ranges were reported. For categorical variables, proportions were calculated. The description was performed overall, separately for men and women, and according to BMI category (normal weight BMI < 25; overweight ≥ 25, <30; obese ≥ 30), and we report the p-value for chi-square or ANOVA test. Pearson correlation coefficients between meal-timing variables were calculated. We used only the data from weekdays, as there were only minimal differences with weekends, with less than an hour difference between the usual time of meals (Table S2 and Figure S2A and S2B), as previous studies [42] had reported that when eating jet lag was of 3.5 h or more, BMI could significantly increase. Furthermore, since they are more frequent, weekdays are more representative of total usual timing, and less susceptible to recall bias compared to weekends, as it has been done in previous studies. [43].

We conducted cross-sectional and longitudinal analyses of the associations between individual variables of meal timing, and BMI. The outcome BMI was assessed in 2018 at the same time as the meal/sleep timing variables (cross-sectional analysis, *n* = 7074), and again in 2023 in a subsample (*n* = 3128, longitudinal analysis). We developed a DAG (Figure S3) to determine which covariates to include in the multivariate analysis. Multiple linear regression models were used to assess the association between the three following variables: number of eating occasions, time of first meal, and nighttime fasting duration, with BMI (continuous outcome, increment of 1 unit). Beta coefficients (β) and 95% confidence intervals (95% CI) were estimated. A main set of covariates was used: age (continuous, years), sex (women/men; except when stratifying by sex), education (university or higher education: yes/no, as other categorizations (primary education, secondary education) ended up with too few individuals in some categories), employment (employed, unemployed), smoking habits (smoker, former smoker and never smoker), alcohol consumption (continuous, grams/week), physical activity (continuous, METs/week), Mediterranean diet score (low, medium or high adherence), energy intake (continuous, average daily kcal), and mental health (poor mental health defined as < 60% in the Mental Health Inventory score: yes/no). We first individually examined the association of each of the 3 meal timing variables in 3 separate models (Model 0). Then, in Model 1 and 2, the meal timing variables were mutually adjusted for (i.e., included in the same model). Model 1 used the same covariates as Model 0, whereas Model 2 further included sleep dissatisfaction and sleep time (continuous, time in hours). In the longitudinal analysis, regressions models were also adjusted for BMI in 2018, to model the *change* in BMI between 2018 and 2023. We tested for multicollinearity by calculating the variance inflation factors (VIF), using a threshold of VIF > 10 to detect presence of multicollinearity.

We examined the interaction between nighttime fasting duration and time of first meal [35] by using the likelihood ratio test between nested models without and with the interaction term and stratified by time of first meal. We assessed this interaction because, the overnight fasting periods might influence metabolic processes (insulin sensitivity, lipid metabolism, among others). Moreover, it would not be the same to have an overnight fast of 12 h starting it at 11PM and finishing it a 11AM than starting it at 7:30PM and finishing it at 7:30AM. Therefore, we assessed this interaction to explore potential synergistic effects on metabolic health of duration and timing of the overnight fast, to get a more comprehensive understanding of the relationship between chrono-nutritional behaviors and adiposity. We also stratified the analyses by sex and menopausal status. Furthermore, we created models without adjusting for all meal timing variables to see the impact of mutual adjustment.

Cluster analysis was performed to group participants with similar meal timing schedules. For the creation of the clusters, variables were standardized, and Gower distance and k-medoids algorithm were used to group individuals with correlated chrono-nutritional variables, by using the R package “cluster” [44]. Gower distance was used because this method can accommodate different types of variables (discrete and continuous). After exploration of correlations (Figure S3) to ensure information used is not redundant, we used the following four variables to create the clusters: nighttime fasting duration, hours between last meal and sleep time, eating midpoint, and number of eating occasions. The number of clusters were selected by using the silhouette method [45]: this method evaluates how effectively data aligns with its assigned cluster compared to other clusters, with a scale from − 1 to 1, where a score of 1 signifies optimal alignment. We selected the value where the silhouette width is maximized at. For selecting the number of values, K value (K = 3) was used and has been added to the text accordingly. Values of K = 2 were also tested, but optimal alignment was not reached and two clusters for each sex were not as clear in terms of variable results when compared to K = 3. Clusters were derived separately for men and women, and characteristics across clusters were described. As men and women have biological and physiological differences, especially hormonal differences, knowing that the circadian system is influenced by this, and that gender roles might influence meal timing (e.g. only women are “housewives”, different occupations, work hours, caring responsibilities, etc.), we decided to explore it separately. To explore the relationship between the clusters and the outcome BMI as a continuous variable, multivariable linear regressions were used, including as covariates the same variables described in model 1 (except sex, as clusters are sex-specific).

All statistical analyses were performed using R studio version 4.2.2, statistical tests were considered significant when *p* < 0.05.

## Results

Our sample for the cross-sectional analysis consisted of 7074 participants, 4166 (58.9%) were women, the average age was 50.8 (SD = 7.0) years, and the overall median BMI 26.2 (IQR [23.8,29.3]) kg/m^2^ (Table 1). Compared to men, women were more likely to have a high adherence to a Mediterranean diet, less likely to consume alcohol, had poorer mental health, a higher percentage of overseeing the house or family, and a lower BMI. Compared to participants with a normal weight, participants with obesity (21.0%) had a lower Mediterranean diet score, were more likely to be in a situation of unemployment, to have a later chronotype, and a poorer mental health. Participants with obesity had a later median time of first meal (8:30 h, IQR [7:30,9:30]) than participants with normal BMI and overweight, (8:00 h (IQR [7:15,9:00]) and 8:00 h (IQR [7:30,9:15]), respectively) and had the longest nighttime fasting duration (11:15 h, IQR [10:00, 12:30]).

**Table 1:** Description of the study population, GCAT study, Catalonia, Spain (*N* = 7,074)

	Overall	Strata: Sex *n* (%)				Strata: BMI			
		Women	Men	*p*	normal		overweight	obese	*p*
**n**	7074	4166	2908		2632		2987 (42.2%)	1455 (20.5%)	
**Age (mean (SD))**	50.80 (7.01)	50.25 (6.81)	51.59 (7.23)	< 0.001	49.27 (6.94)		51.56 (6.96)	52.02 (6.77)	< 0.001
**Sex (men)**	2908 (41.1)					738 (28.0)	1502 (50.3)	668 (45.9)	< 0.001
**Menopause (%)**		2473 (59.3)				1007 (53.1)	971 (65.3)	495 (63.0)	< 0.001
**rMEDScore (%)** ^**1**^				< 0.001					< 0.001
Low adherence	1631 (23.1)	761 (18.3)	870 (29.9)		547 (20.8)		706 (23.6)	378 (26.0)	
Medium adherence	3418 (48.3)	1952 (46.9)	1466 (50.4)		1231 (46.8)		1469 (49.2)	718 (49.3)	
High adherence	2025 (28.6)	1453 (34.9)	572 (19.7)		854 (32.4)		812 (27.2)	359 (24.7)	
**Energy intake (kcal/day) (mean (SD))**	1996.46 (586.57)	1940.46 (563.73)	2076.68 (609.01)	< 0.001	1987.93 (575.13)		1996.24 (585.00)	2012.34 (609.90)	0.444
**High education**	3538 (50.0)	2095 (50.3)	1443 (49.6)	0.598	1490 (56.6)		1416 (47.4)	632 (43.4)	< 0.001
**Employment status**				< 0.001					< 0.001
Paid or self-employed	5508 (78.9)	3224 (78.3)	2284 (79.7)		2162 (83.0)		2284 (77.7)	1062 (73.8)	
Retired	496 (7.1)	205 (5.0)	291 (10.2)		114 (4.4)		251 (8.5)	131 (9.1)	
In charge of the house/family	253 (3.6)	240 (5.8)	13 (0.5)		92 (3.5)		102 (3.5)	59 (4.1)	
Unemployment	724 (10.4)	446 (10.8)	278 (9.7)		236 (9.1)		301 (10.2)	187 (13.0)	
**Chronotype**				< 0.001					0.003
Clearly morning	1895 (27.4)	1059 (25.9)	836 (29.4)		743 (28.8)		805 (27.5)	347 (24.4)	
More morning than evening	2412 (34.8)	1378 (33.7)	1034 (36.4)		887 (34.4)		1055 (36.1)	470 (33.1)	
More evening than morning	1921 (27.7)	1175 (28.8)	746 (26.3)		691 (26.8)		787 (26.9)	443 (31.2)	
Clearly evening	698 (10.1)	474 (11.6)	224 (7.9)		261 (10.1)		276 (9.4)	161 (11.3)	
**Mental health score: poor mental health (< 60%)** ^**2**^	1907 (27.0)	1293 (31.0)	614 (21.1)	< 0.001	696 (26.4)		764 (25.6)	447 (30.7)	0.001
**Alcohol g/d (mean (SD))**	8.05 (10.87)	5.38 (7.31)	11.88 (13.64)	< 0.001	6.83 (8.70)		8.78 (11.04)	8.77 (13.52)	< 0.001
**Smoking habit (%)**				0.051					< 0.001
Current	1033 (14.6)	644 (15.5)	389 (13.4)		398 (15.1)		427 (14.3)	208 (14.3)	
Former-smoker	3151 (44.5)	1836 (44.1)	1315 (45.2)		1087 (41.3)		1348 (45.1)	716 (49.2)	
Never	2890 (40.9)	1686 (40.5)	1204 (41.4)		1147 (43.6)		1212 (40.6)	531 (36.5)	
**Physical activity (METS/wk) (median [IQR])**	61.54 [39.67, 97.05]	62.12 [40.81, 94.39]	60.90 [36.89, 99.51]	0.037	65.30 [42.59, 99.94]		62.67 [40.23, 98.83]	53.99 [32.15, 86.76]	< 0.001
**Breakfast consumption (%)**	6853 (96.9)	4082 (98.0)	2771 (95.3)	< 0.001	2559 (97.2)		2891 (96.8)	1403 (96.4)	0.346
**First meal before 8:30 h (%)**	4236 (59.9)	2526 (60.6)	1710 (58.8)	0.128	1676 (63.7)		1765 (59.1)	795 (54.6)	< 0.001
**Time of first meal (median [IQR])**	8:00 [7:19, 9:30]	8:00 [7:15, 9:19]	8:00 [7:30, 9:30]	0.115	8:00 [7:15, 9:00]		8:00 [7:30, 9:15]	8:30 [7:30, 9:30]	< 0.001
**Time of last meal (mean (SD))**	21:08 (0:45)	21:07 (0:45)	21:10 (0:46)	0.011	21:06 (0:43)		21:14 (0:48)	21:08 (0:45)	< 0.001
**Fasting hours (median [IQR])**	11:00 [10:00, 12:00]	11:00 [10:00, 12:10]	11:00 [10:00, 12:00]	0.874	11:00 [10:00, 12:00]		11:00 [10:00, 12:00]	11:15 [10:00, 12:30]	0.067
**Hours between wake up and first meal (median [IQR] )**	1:00 [0:30, 2:30]	1:00 [0:25, 2:30]	1:00 [0:30, 2:30]	0.001	0:55 [0:20, 2:30]		1:00 [0:30, 2:30]	1:00 [0:30, 2:30]	0.002
**Hours between last meal and sleep (mean (SD))**	2:15 (0:53)	2:14 (0:52)	2:17 (0:54)	0.025	2:15 (0:48)		2:16 (1:00)	2:16 (0:53)	0.639
**Eating midpoint (mean (SD))**	14:49 (0:57)	14:47 (0:55)	14:52 (0:59)	< 0.001	14:44 (0:56)		14:55 (0:58)	14:59 (0:56)	< 0.001
**Number of eating occasions (mean (SD))**	3.42 (0.60)	3.49 (0.59)	3.31 (0.59)	< 0.001	3.44 (0.59)		3.43 (0.62)	3.40 (0.59)	0.033
**Wake-up time (mean (SD))**	6:57 (0:55)	6:58 (0:54)	6:56 (0:56)	0.09	6:55 (0:52)		7:00 (1:00)	6:58 (0:55)	0.007
**Sleep time (mean (SD))**	23:30 (0:50)	23:27 (0:50)	23:34 (0:50)	< 0.001	23:26 (0:46)		23:35 (0:58)	23:30 (0:50)	< 0.001
**Sleep dissatisfaction** ^**3**^ **(%)**	964 (13.6)	626 (15.0)	338 (11.6)	< 0.001	355 (13.5)		386 (12.9)	223 (15.3)	0.087
**Sleep duration in hours (mean (SD))**	7:27 (0:54)	7:30 (0:53)	7:22 (0:55)	< 0.001	7:28 (0:51)		7:26 (0:59)	7:27 (0:55)	0.342
**BMI** ^**4**^ **(median [IQR])**	26.23 [23.78, 29.30]	25.43 [23.09, 28.80]	27.06 [24.95, 29.71]	< 0.001	23.11 [21.83, 24.09]		27.08 [26.02, 28.40]	32.68 [31.09, 35.08]	

N, sample size; SD, standard deviation; IQR, interquartile range

1. rMED ranges from 0 to 18. Adherence levels were classified into: low (0–6), medium (7–10), and high (11–18)

2.Poor mental health is defined as a Mental Health Inventory score < 60%

3. Sleep dissatisfaction: frequency of participants that answer “no” to the question “are you satisfied with your sleep?”

4. BMI, body mass index

### Cross-sectional associations

In cross-sectional analysis (Table 2), when not mutually adjusted, only a later time of first meal was associated with a higher BMI (β = 0.06; 95% CI 0.00,0.12). When put together in a model, a later time of first meal showed a stronger association with a higher BMI (β = 0.32; 95% CI 0.18,0.47), while a longer nighttime fasting duration was associated with a lower BMI (β= -0.27; 95% CI -0.41, -0.13). When further adjusting for sleep time and sleep dissatisfaction, results were slightly attenuated. In men, only the association with time of first meal was statistically significant (β = 0.23; 95% CI 0.04,0.43), and it was attenuated when adjusting the model for sleep variables. In women, the same pattern was observed as in the whole population, and additionally more eating occasions (β = 0.25, 95% CI 0.00, 0.51) and later sleep time (β = 0.20; 95% CI 0.01, 0.39) showed an association with higher BMI. In postmenopausal women, only a higher number of eating occasions was associated with a higher BMI (β = 0.47, 95% CI 0.16, 0.78). In premenopausal women, time of first meal (β = 0.58, 95% CI 0.25, 0.90), and nighttime fasting hours (β=-0.59, 95% CI -0.91, -0.28) were associated with BMI and these associations were attenuated when adjusting for sleep variables, with sleep dissatisfaction (β = 0.77, 95% CI 0.09, 1.45) and a later sleep time (β = 0.31, 95% CI -0.01, 0.62) being associated with higher BMI. Regarding multicollinearity, all generalized VIF values < 10, indicating little evidence of collinearity, although some collinearity was observed for nighttime fasting and time of first meal (Table S3A and S3B).

**Table 2:** Multiple linear regression models assessing the relationship between meal and sleep timing variables and body mass index: cross-sectional analysis (GCAT study 2018)

		Exposures^1^				
		Time of first meal	Fasting hours	Eating occasions	Sleep dissatisfaction	Sleep time
Overall (*n* = 7074)						
	Model 0^2^	**0.06 [0.00**,** 0.12]**	0.00 [-0.06, 0.06]	0.16 [-0.02, 0.33]		
	Model 1^3^	**0.32 [0.18**,** 0.47]**	**-0.27 [-0.41**,** -0.13]**	0.14 [-0.05, 0.32]		
	Model 2^4^	**0.24 [0.08**,** 0.40]**	**-0.19 [-0.35**,** -0.04]**	0.15 [-0.04, 0.33]	0.19 [-0.11, 0.50]	**0.16 [0.02**,** 0.29]**
Men (*n* = 2908)						
	Model 1^3^	**0.23 [0.04**,** 0.43]**	-0.18 [-0.37, 0.01]	-0.01 [-0.27, 0.26]		
	Model 2^4^	0.18 [-0.03, 0.4]	-0.14 [-0.34, 0.07]	0.00 [-0.26, 0.27]	0.39 [-0.06, 0.84]	0.1 [-0.09, 0.28]
Women (*n* = 4166)						
	Model 1^3^	**0.39 [0.19**,** 0.59]**	**-0.33 [-0.53**,** -0.13]**	0.24 [-0.01, 0.49]		
	Model 2^4^	**0.28 [0.05**,** 0.50]**	**-0.24 [-0.45**,** -0.02]**	**0.25 [0.00**,** 0.51]**	0.10 [-0.30, 0.50]	**0.20 [0.01**,** 0.39]**
Premenopausal women (*n* = 1693)						
	Model 1^3^	**0.58 [0.25**,** 0.90]**	**-0.59 [-0.91**,** -0.28]**	-0.09 [-0.50, 0.33]		
	Model 2^4^	**0.40 [0.04**,** 0.77]**	**-0.45 [-0.80**,** -0.11]**	-0.07 [-0.49, 0.34]	**0.77 [0.09**,** 1.45]**	0.31 [-0.01, 0.62]
Postmenopausal women (*n* = 2473)						
	Model 1^3^	0.25 [-0.01, 0.51]	-0.12 [-0.37, 0.13]	**0.47 [0.16**,** 0.78]**		
	Model 2^4^	0.18 [-0.1, 0.47]	-0.06 [-0.34, 0.21]	**0.48 [0.18**,** 0.80]**	-0.25 [-0.75, 0.25]	0.13 [-0.11, 0.37]

N: sample size. **Bold: statistically significant**

1. Values are regression coefficients [95% confidence interval]. Coefficients should be interpreted for 1-hour increase for each time exposure, for eating occasions it should be interpreted as an increase in 1 eating occasion, for sleep dissatisfaction, it should be interpreted as a higher degree of dissatisfaction. Outcome: BMI. Multicollinearity test was performed, all GVIF < 10.

2. Model 0: Meal timing variables not mutually adjusted, i.e. three separate models. Adjustment variables: age, sex, adherence to Mediterranean diet, education, employment, mental health, smoking habit, physical activity, time of first meal, fasting hours, number of eating occasions, and energy intake

3. Model 1 same adjustment variables. Meal timing variables mutually adjusted in one sole model

4. Model 2 includes all the variables in Model 1 + sleep dissatisfaction and sleep time

There was a significant interaction between time of first meal and fating duration, and when stratifying (Table S4), we found that associations with time of first meal and fasting duration were only apparent for people who have their first meal after 8:30, particularly in women.

### Longitudinal associations

For these analyses, the sample size was restricted (*n* = 3128), 40% were men, the mean age was 50.9 years (Table S5) and participants had overall healthier habits than those in the cross-sectional sample. Average median BMI decreased by 0.26 kg/m^2^ between 2018 and 2023. The associations observed in longitudinal analyses (Table 3) were similar to the cross-sectional associations, particularly in men. Longer fasting hours were associated with a lower change in BMI (β=-0.11, 95% CI -0.21, -0.01); this relationship was maintained when adjusting for sleep, and a later time of first meal was associated with a greater BMI change (β = 0.12; 95% CI 0.00,0.24). In women, the associations were weaker and were not statistically significant. When stratifying these results by time of first meal (Table S6), associations were only apparent for people who have their first meal after 8:30, particularly in men.

**Table 3:** Multiple linear regression models assessing the relationship between meal and sleep timing variables and change in body mass index: longitudinal analysis (GCAT study 2018–2023)

		Exposures ^1^					
		Time of first meal	Fasting hours	Eating occasions	Sleep dissatisfaction	Sleep time	
Overall (*n* = 3128)							
	Model 0 ^2^	-0.01 [-0.06, 0.04]	-0.03 [-0.08, 0.01]	0.04 [–0.09, 0.17]			
	Model 1 ^3^	0.09 [-0.01, 0.20]	**-0.11 [-0.21**,** -0.01]**	0.00 [-0.13, 0.14]			
	Model 2 ^4^	**0.12 [0.00**,** 0.24]**	**-0.13 [-0.24**,** -0.02]**	0.00 [-0.14, 0.14]	0.09 [-0.15, 0.32]	-0.05 [-0.15, 0.05]	
Men (*n* = 1238)							
	Model 1	**0.15 [0.00**,** 0.29]**	**-0.17 [-0.31**,** -0.02]**	-0.17 [-0.37, 0.04]			
	Model 2	**0.18 [0.02**,** 0.34]**	**-0.19 [-0.35**,** -0.04]**	-0.18 [-0.38, 0.03]	0.19 [-0.18, 0.55]	-0.07 [-0.21, 0.07]	
Women (*n* = 1890)							
	Model 1	0.05 [-0.09, 0.20]	-0.08 [-0.22, 0.06]	0.09 [-0.09, 0.27]			
	Model 2	0.07 [-0.09, 0.24]	-0.10 [-0.25, 0.06]	0.09 [-0.10, 0.27]	0.04 [-0.27, 0.34]	-0.04 [-0.18, 0.10]	
Premenopausal women (*n* = 797)							
	Model 1	-0.05 [-0.28, 0.19]	0.04 [-0.19, 0.28]	0.22 [-0.09, 0.52]			
	Model 2	0.03 [-0.23, 0.29]	-0.02 [-0.27, 0.23]	0.20 [-0.10, 0.51]	0.16 [-0.35, 0.68]	-0.15 [-0.37, 0.08]	
Postmenopausal women (*n* = 1093)							
	Model 1	0.12 [-0.06, 0.31]	-0.17 [-0.35, 0.01]	0.00 [-0.23, 0.23]			
	Model 2	0.09 [-0.12, 0.30]	-0.14 [-0.34, 0.06]	0.01 [-0.22, 0.24]	-0.07 [-0.45, 0.31]	0.06 [-0.12, 0.23]	

N: sample size. **Bold: statistically significant**

1. Values are regression coefficients [95% confidence interval]. Coefficients should be interpreted for 1-hour increase for each time exposure, for eating occasions it should be interpreted as an increase in 1 eating occasion, for sleep dissatisfaction, it should be interpreted as a higher degree of dissatisfaction. Outcome: BMI. Multicollinearity test was performed, all GVIF < 10.

2. Model 0: Meal timing variables not mutually adjusted, i.e. three separate models. Adjustment variables: age, sex, adherence to Mediterranean diet, education, employment, mental health, smoking habit, physical activity, time of first meal, fasting hours, number of eating occasions, energy intake, and baseline BMI

3. Model 1 : same adjustment variables. Meal timing variables mutually adjusted in one sole model

4. Model 2 includes all the variables in Model 1 + sleep dissatisfaction and sleep time

### Meal timing patterns

**Fig. 1:**
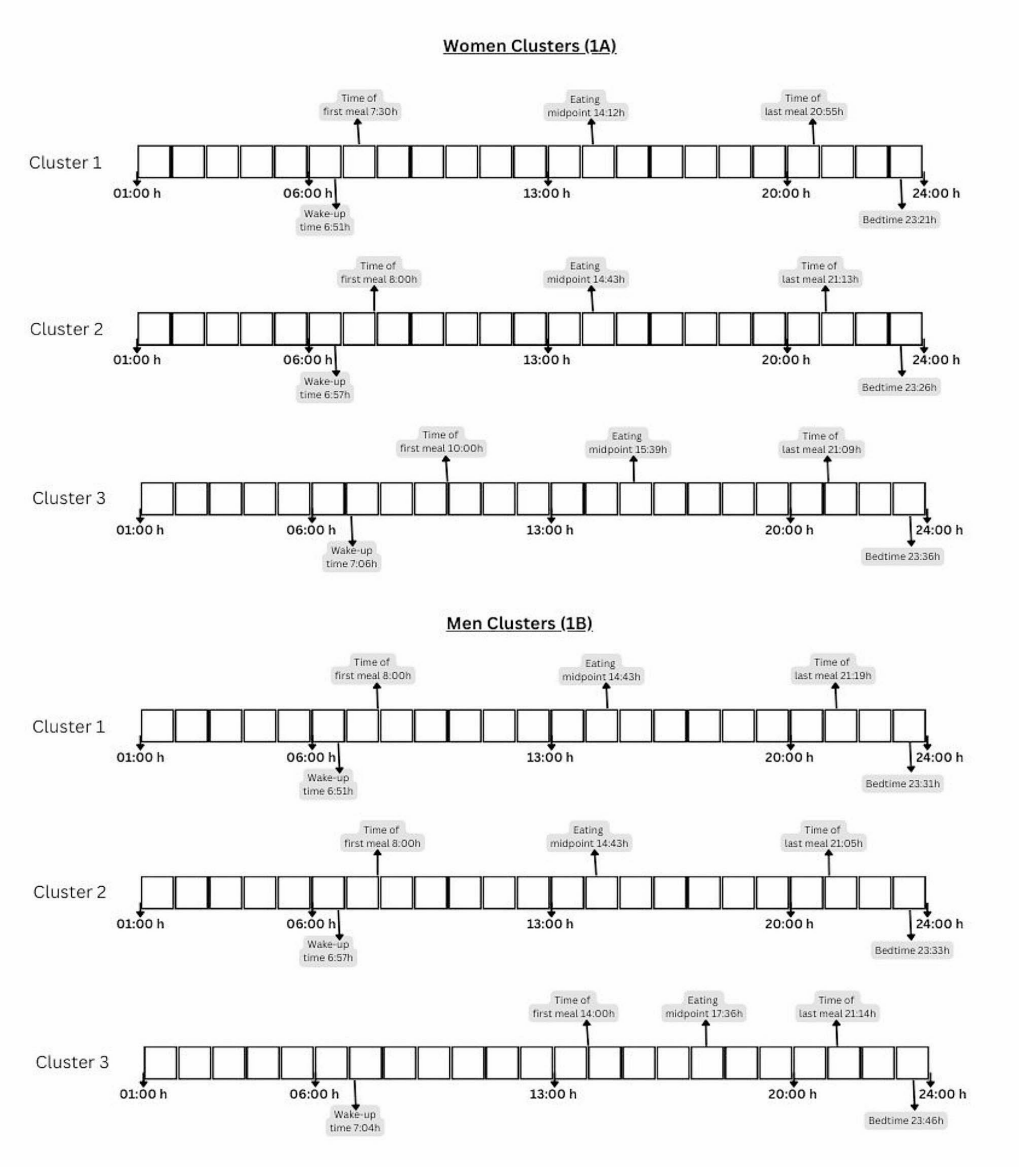
Meal and sleep timing variables in women (1A) and men (1B) chrono-nutritional clusters

When analyzing the silhouette width for men, the optimal number of clusters were 3; even though for women the optimal number of clusters was 2, we chose the second best option, 3, because it made the differences between the eating patterns across women clusters clearer and made it more comparable with the men-specific analysis (Figure S4). Three clusters were obtained (K = 3), separately for men and women (Table 4; Fig. 1). In women **(**Fig. 1A), clusters differed mostly according to the time of first meal and number of eating occasions. Cluster 1 (*n* = 1,233, 29.5% of the sample) was characterized by the earliest median time of first meal (7:30 h, IQR [7:00, 8:00]) and eating midpoint (mean = 14:12 h; SD = 0:30 h), the shortest nighttime fasting duration (10:30 h, IQR [10:00, 11:00]) and by having 3 eating occasions. This cluster had the highest Mediterranean diet score, the greatest percentage of people with high education and in employment, the lowest frequency of poor mental health, and the lowest BMI (25.2 kg/m^2^). Cluster 2 (*n* = 1,986, 47.6%) was formed by women habitually having 4 eating occasions, who were younger, had higher levels of physical activity, and they had the latest time of last meal (21:14, SD = 0:48 h) and a median BMI of 25.6 kg/m^2^. Cluster 3 (*n* = 947, 22.9%) was characterized by the latest median time of first meal (10:00 h, IQR [9:00, 10:30]), 3 eating occasions, the lowest adherence to a Mediterranean diet and the highest percentage of unemployed and housewives (6.8%), had poorer mental health, and a later chronotype.

**Table 4:** Main characteristics of the chrono-nutritional clusters stratified by sex (GCAT study, *N* = 7074)

	Women				Men			
	Cluster 1	Cluster 2	Cluster 3	*p*	Cluster 1	Cluster 2	Cluster 3	*p*
**n**	1,233 (29.5%)	1,986 (47.6%)	947 (22.9%)		949 (32.6%)	1,822 (62.7%)	137 (4.7%)	
**Age (mean(SD))**	50.25 (6.66)	49.91 (6.72)	50.97 (7.12)	< 0.001	50.75 (7.37)	52.12 (7.10)	50.36 (7.25)	< 0.001
**Menopause (%)**	732 (59.4)	1147 (57.8)	591 (62.5)	0.052				
**rMEDScore (%)** ^**1**^				0.024				0.034
Low adherence	201 (16.3)	356 (17.9)	204 (21.5)		275 (29.0)	542 (29.7)	53 (38.7)	
Medium adherence	583 (47.3)	931 (46.9)	438 (46.3)		480 (50.6)	916 (50.3)	70 (51.1)	
High adherence	449 (36.4)	699 (35.2)	305 (32.2)		194 (20.4)	364 (20.0)	14 (10.2)	
**Energy intake (kcal/day)**	1,915.22 (539.51)	1,990.71 (578.72)	1,867.94 (553.09)	<0.001	2,159.99 (623.34)	2,047.23 (599.16)	1,891.22 (563.67)	< 0.001
**Education (university or higher) (%)**	711 (57.7)	982 (49.4)	402 (42.4)	< 0.001	447 (47.1)	936 (51.4)	60 (43.8)	0.039
**Employment status (%)**				0.012				0.005
Paid or self-employed	980 (80.5)	1531 (78.2)	713 (76.0)		744 (79.3)	1435 (80.1)	105 (77.2)	
Retired	55 (4.5)	85 (4.3)	65 (6.9)		84 (9.0)	199 (11.1)	8 (5.9)	
In charge of the house/family	57 (4.7)	119 (6.1)	64 (6.8)		3 (0.3)	9 (0.5)	1 (0.7)	
In a situation of unemployment	126 (10.3)	224 (11.4)	96 (10.2)		107 (11.4)	149 (8.3)	22 (16.2)	
**Chronotype (%)**				< 0.001				0.02
Clearly morning	356 (29.3)	516 (26.4)	187 (20.3)		305 (32.9)	495 (27.8)	36 (27.3)	
More morning than evening	437 (36.0)	660 (33.8)	281 (30.5)		327 (35.3)	667 (37.4)	40 (30.3)	
More evening than morning	308 (25.4)	545 (27.9)	322 (35.0)		223 (24.1)	484 (27.2)	39 (29.5)	
Clearly evening	112 (9.2)	231 (11.8)	131 (14.2)		71 (7.7)	136 (7.6)	17 (12.9)	
**Mental health score: poor mental health (< 60%)** ^**2**^	361 (29.3)	614 (30.9)	318 (33.6)	0.097	190 (20.0)	373 (20.5)	51 (37.2)	< 0.001
**Alcohol g (mean (SD))**	6.01 (7.41)	4.48 (6.48)	6.45 (8.52)	< 0.001	8.76 (10.97)	13.28 (14.39)	14.77 (16.18)	< 0.001
**Smoking habit (%)**				< 0.001				< 0.001
Current	185 (15.0)	258 (13.0)	201 (21.2)		101 (10.6)	255 (14.0)	33 (24.1)	
Ex-smoker	538 (43.6)	909 (45.8)	389 (41.1)		421 (44.4)	830 (45.6)	64 (46.7)	
Never	510 (41.4)	819 (41.2)	357 (37.7)		427 (45.0)	737 (40.5)	40 (29.2)	
**Physical activity [METS/week] (median (IQR))**	60.8 [40.4, 90.8]	65.0 [42.6, 102.0]	60.1 [36.3, 87.2]	< 0.001	67.3 [41.8, 107.3]	58.7 [34.7, 96.8]	53.3 [27.8, 87.5]	< 0.001
**Breakfast consumption (%)**	1233 (100.0)	1985 (99.9)	864 (91.2)	< 0.001	946 (99.7)	1814 (99.6)	11 (8.0)	< 0.001
**First meal before 8:30 h (%)**	1223 (99.2)	1296 (65.3)	7 (0.7)	< 0.001	625 (65.9)	1085 (59.5)	0 (0.0)	< 0.001
**Time of first meal (median [IQR])**	7:30 [7:00, 8:00]	8:00 [7:01, 9:00]	10:00 [9:00, 10:30]	< 0.001	8:00 [7:00, 9:00]	8:00 [7:30, 9:00]	14:00 [13:30, 14:30]	< 0.001
**Time of last meal (mean (SD))**	20:56 (0:40)	21:14 (0:49)	21:10 (0:40)	< 0.001	21:19 (0:53)	21:05 (0:38)	21:14 (1:00)	< 0.001
**Fasting hours (median [IQR]**	10:30 [10:00, 11:00]	11:00 [10:00, 12:00]	12:30 [12:00, 13:30]	< 0.001	10:45 [10:00, 12:00]	11:00 [10:15, 12:00]	17:00 [16:00, 17:30]	< 0.001
**Hours between wake up and first meal (median [IQR])**	0:30 [0:15, 1:00]	0:45 [0:15, 2:00]	3:00 [2:00, 4:00]	< 0.001	0:45 [0:20, 2:00]	1:00 [0:30, 2:30]	7:00 [6:00, 7:30]	< 0.001
**Hours between last meal and sleep onset (mean (SD))**	2:20 (0:49)	2:08 (0:53)	2:20 (0:50)	< 0.001	2:06 (0:59)	2:22 (0:50)	2:25 (1:03)	< 0.001
**Eating midpoint (mean (SD))**	14:13 (0:30)	14:43 (0:50)	15:39 (0:50)	< 0.001	14:44 (0:52)	14:44 (0:44)	17:36 (0:44)	< 0.001
**Number of eating occasions (mean (SD))**	2.99 (0.08)	4.07 (0.26)	2.94 (0.24)	< 0.001	4.07 (0.26)	2.99 (0.08)	2.20 (0.42)	< 0.001
**BMI** ^**3**^ **(median [IQR])**	25.15 [22.95, 28.51]	25.63 [23.18, 29.00]	25.47 [23.08, 28.77]	0.032	26.98 [24.78, 29.46]	27.07 [25.07, 29.74]	27.53 [25.14, 30.11]	0.153
**Wake-up time (mean (SD))**	6:52 (0.75)	6:58 (0.91)	7:07 (1.04)	< 0.001	6:52 (0.94)	6:57 (0.91)	7:05 (1.20)	0.007
**Sleep time (mean (SD))**	23:22 (0:49)	23:26 (0:49)	23:36 (0:54)	< 0.001	23:31 (0:50)	23:34 (0:49)	23:46 (1:03)	0.006
**Sleep dissatisfaction** ^**3**^ **(%)**	160 (13.0)	307 (15.5)	159 (16.8)	0.036	112 (11.8)	209 (11.5)	17 (12.4)	0.926
**Sleep duration in hours (mean (SD))**	7:30 (0:52)	7:31 (0:52)	7:31 (0:58)	0.779	7:20 (0:55)	7:23 (0:54)	7:19 (1:07)	0.301

N, sample size; SD, standard deviation; IQR, interquartile range

1. rMED ranges from 0 to 18. Adherence levels were classified into: low (0–6), medium (7–10), and high (11–18)

2.Poor mental health is defined as a Mental Health Inventory score < 60%

3. Sleep dissatisfaction: frequency of participants that answer “no” to the question “are you satisfied with your sleep?”

In men (Fig. 1B), cluster 1 (*n* = 949, 32.6%) was characterized by having 4 eating occasions, the earliest time of first meal (8:00 h, IQR [7:00, 9:00]) and shortest nighttime fasting duration (10:45 h, IQR [10:00, 12:00]). The mean age was 50.8 years, they were more likely to have a high Mediterranean diet score, a morning chronotype, be non-smoker, and had the lowest mean consumption of alcohol. In cluster 2 (*n* = 1,822, 62.7%), almost 100% of men consumed breakfast, the time of the last meal was the earliest in comparison to the other two clusters (21:05 h, SD = 0:38 h), and the median of eating occasions was 3. Cluster 3 was small (*n* = 137, 4.7%) and was characterized by men having a much later median time of first meal (14:00 h, IQR [13:30, 14:30]) and mean eating midpoint (17:36 h, SD = 0:44 h), 92% reported skipping breakfast and their median nighttime fasting duration was 17:00 h, and they consumed only 2 eating occasions. Men in this cluster were more likely to have a lower education, be unemployed, have a low Mediterranean diet score, have poorer mental health and higher alcohol and tobacco consumption, and reported a more evening chronotype.

Compared to cluster 1 (Table 5), women in cluster 3 had a significantly higher BMI (beta = 0.33, 95% CI 0.01–0.66). In men, we did not find any statistically significant associations across clusters.

**Table 5:** Multivariate linear regressions models ^a^ assessing the relationship between chrono-nutritional clusters with body Mass Index (GCAT study, *N* = 7074)

Outcome	Women			Men		
	Cluster 1 (*N* = 1233)	Cluster 2 (*N* = 1986)	Cluster 3 (*N* = 947)	Cluster 1 (*N* = 949)	Cluster 2 (*N* = 1822)	Cluster 3 (*N* = 137)
**BMI continuous** **(β [95% CI])**	REF	0.04 [-0.35, 0.44]	**0.33 [0.01**,** 0.66]**	REF	0.07 [-0.23, 0.38]	0.24 [-0.46, 0.93]

N = sample size. Coefficients should be interpreted as BMI (kg/m^2^) change in comparison to cluster 1

**Bold: statistically significant.**^**a**^ Values are regression coefficients [95% confidence interval]. Model includes the following variables: age, adherence to Mediterranean diet, education, employment, mental health, smoking habit, physical activity, energy intake

## Discussion

In this population-based cohort of adults in Catalonia, a later time of first meal, more daily eating occasions, and a later sleep time were cross-sectionally related to a higher BMI, while a longer nighttime fasting duration was related to a lower BMI. Among women, these results were mostly evident in premenopausal women. In longitudinal analyses, a later time of first meal and a shorter nighttime fasting duration were associated with a higher subsequent BMI. Finally, cluster analysis revealed groups with different meal-timing patterns, particularly differentiated by the time of first meal and the number of eating occasions, that were cross-sectionally associated with different sociodemographic, lifestyle and health profiles.

### Meal timing and adiposity

There is a growing body of animal and human studies on the relationship between meal timing and adiposity. One study [46] that created a delayed meal timing protocol for rats (4-hour delay compared to controls), suggested that energy expenditure was decreased in response to a later first meal and concluded that breakfast skipping may exacerbate fatty liver and body fat accumulation. Another rat model [47] found that early nocturnal fasting, mimicking “breakfast skipping” as rats are nocturnal animals, disturbs the peripheral clock and increases lipid synthesis, increasing predisposition to obesity. In humans, a cohort from US and Canada [48] revealed that having fewer eating occasions, consuming breakfast, and eating the largest meal in the morning may be effective methods for preventing long-term weight gain, and that a long overnight fast was associated with a lower long-term BMI gain. Similarly, an Italian population study [49] showed that individuals eating in a time window limited to 8–10 h during the day (i.e. with a longer nighttime fasting duration), were less likely to be obese and overweight. In a randomized cross-over nutritional intervention study [50], meal skipping increased energy expenditure and disturbed glucose homeostasis, but data did not support a causal role of breakfast skipping in the development of obesity. A meta-analysis [26] reported that breakfast skipping is associated with an increased risk of overweight and obesity, with the results being consistent for different ages, genders, regions, and economic conditions. A genome-wide association study of breakfast skipping [51] reported the involvement of six genetic variants in this behavior and found a link between clock regulation and food timing. Moreover, they observed that genetically defined breakfast skipping was associated with a higher BMI. Our findings are consistent with the conclusions of these studies, also suggesting a relationship between an earlier time of first meal and a lower BMI. Conversely, a systematic review and meta-analysis of seven randomized trials [15] suggested that skipping breakfast could potentially lead to modest weight loss while possibly raising short-term LDL-cholesterol levels. In our independent models, a later time of first meal, but not nighttime fasting duration, was associated with a higher BMI. When mutually adjusting both meal timing variables, a longer nighttime fasting duration was associated with a lower BMI, suggesting that the deleterious impact of delaying the time of first meal was masking the beneficial association of prolonging the nighttime fasting duration. Including different meal timing variables in the same model is important to grasp the full picture of the associations, and is becoming standard practice in chrono-nutritional studies [52–54]. However, long-term interventions are necessary to draw definitive conclusions.

An important aspect of our results is that we adjusted for adherence to a Mediterranean diet, a marker of diet quality. The associations between meal timing variables and BMI were independent both of diet quality and caloric intake, as we adjust models for both variables separately. This was important as cluster analysis revealed associations between meal-timing and diet quality. We expected that the beneficial association of longer fasting duration on BMI would be stronger in those who consume an early breakfast (and therefore have an early dinner), but we observed the opposite: extended nighttime fasting hours were found to be beneficial for those who had a later time of first meal. This might be due to the observed low variability in dinner time, and therefore less scope for varying fasting duration in participants who have their first meal before 8:30. A randomized clinical weight loss trial [55] found that early time-restricted eating was more effective for weight loss than eating over a window of 12 or more hours; furthermore, a cross-sectional study [56] concluded that starting energy consumption earlier in the day is beneficial for cardiometabolic outcomes.

We performed a simple form of mediation analysis, which is to assess the attenuation of the coefficients between a model without and with the sleep variables (sleep time and sleep dissatisfaction). The observed attenuation might suggest a mediating effect of sleep dissatisfaction and a later sleep time in the relationship between meal-timing patterns and an increase in BMI, but this may also suggest confounding, as disrupted sleep habits may also lead to mistimed diets. Previous studies [47] have suggested that last eating or drinking within 4 to 6 h before sleep time confers the highest likelihood of achieving optimal sleep, and as the interval of eating or drinking prior to sleep time increases, the odds of short and long sleep durations (i.e., non-optimal sleep duration) decreases.

### Sex and socioeconomic differences in meal timing patterns

Both in men and women, the cluster with the lowest BMI had a higher percentage of a morning self-reported chronotype and earlier time of first meal. A small group of men were breakfast skippers, had only two eating occasions during the day and a very late eating midpoint, with lower educational level and higher unemployment, more likely to engage in unhealthy behaviors (alcohol, smoking, unhealthy diet), and had worse mental health and sleep satisfaction. The difference in timing of meals found in our Mediterranean population compared to the Austrian study goes in line with the findings [28] that Mediterranean countries have later meal times compared with central and northern European countries, but that study did not find a clear association between meal timing and BMI. The cluster analysis (not sex-specific) in the Austrian population [27] found that the group characterized by breakfast skipping and long fasting hours had higher odds of obesity (although it was not statistically significant). In that study [27], information was gathered through surveys conducted in 2017 and 2020, two clusters (A and B) emerged in each sample, with Cluster A consistently showing more respondents, characterized by a fasting duration of 12–13 h and an eating midpoint between 13:00–13:30 h, while Cluster B participants exhibited longer fasting periods, delayed mealtimes, a higher prevalence of breakfast skipping, and of chronic insomnia and obesity, among other outcomes. Another study in a population with obesity in Norway [57], using dietary and meal timing clusters found an association between consuming the highest energy intake at midnight with higher total energy intake throughout the day, although they did not find correlation with anthropometric measures. Finally, in our study we found a statistically significant association of women from cluster 3 and higher BMI, which goes in line with the results of individual regressions.

Regarding sex and socioeconomic differences in nutritional behaviors, a study [58]investigating the trends in adherence to Mediterranean diet and socioeconomic characteristics among workers in Spain from 2006 to 2017, found that workers who had temporary contracts, and engaged in tobacco and alcohol consumption had lower diet quality scores. These findings align with our study, where we observed in the cluster of men characterized with a very late first meal and only 2 eating occasions, that lower employment rates were concomitant with higher alcohol and tobacco consumption and lower adherence to a Mediterranean diet. In contrast, a study conducted in Canada analyzing trends in socioeconomic inequities in adult diet quality from 2004 to 2015 [59] shows that overall diet quality remained stable in the total population and among women across various socioeconomic indicators; however, men with better socioeconomic conditions consistently maintained or improved their diet quality in comparison to those of lower socioeconomic status. Along the same lines, a repeated cross-sectional survey in the UK [60] reported that women and people with non-manual occupations were more likely to adhere to dietary recommendations and concluded that most dietary inequalities persisted across the three surveys conducted between 1986 and 2012. We find comparable results with women displaying a better diet quality compared to men. Additionally, in women, we saw differences when stratifying by menopausal status, with stronger associations in premenopausal women in the cross-sectional analysis. A systematic review [61] found that estrogens play a leading role in the causes and consequences of female obesity, and that mechanisms involving brain signaling pathways, influenced by ovarian hormones, might contribute to overeating and obesity. A previous study found an association in premenopausal women only between a later time of breakfast and higher breast cancer risk [53].

Regarding direct comparisons by sex in meal timing, the only limited evidence from the literature concerns breakfast skipping. A study in adolescents in Spain [62] found that this behavior was more common in girls, and that it was more prevalent in the most disadvantaged socioeconomic levels (both for boys and girls). Another study in adults from Korea [63] did not find any significant difference in regular breakfast intake between men and women. Our results are different from these studies, as there is a small, but significant, difference in the proportion of breakfast consumption in men and women in the whole sample.

It is important to note that this study, among emerging evidence, suggests longer fasting duration might be only beneficial to maintain a healthy weight when accompanied with consumption of breakfast and early dinner. However, this is only one study from which we cannot draw recommendations. Another implication for chrono-nutrition research is that questionnaires used to assess chrono-nutritional exposures should undergo validation and adaptability to diverse cultural contexts to effectively evaluate dietary habits. Lastly, future research in this field should prioritize conducting cohort studies to assess prospectively the relationship between chrono-nutrition and cardiometabolic outcomes, and the role of sleep as a mediator in this relationship.

### Strengths and limitations

A key strength of this study is its novelty and longitudinal results, that are consistent with the cross-sectional analysis. Furthermore, this is the first study describing sex-specific meal-timing patterns. This study also has a comprehensive assessment of meal and sleep patterns, with detailed adjustment for diet quality, and we used a large, well-characterized, heterogeneous, recent population-based cohort of adults in Catalonia. However, we acknowledge several limitations. Firstly, BMI, meal and sleep timing data were self-reported, which might be subject to misreporting and recall bias. Furthermore, we only considered weekday data, and although it was highly correlated with weekend times, further exploration is needed to assess the differences between weekday and weekend eating patterns and the exploration of eating jetlag, as previous studies [5] report that individuals who showed higher eating jet lag also showed greater BMI. Our questionnaire only records information about five eating occasions during the day, with no question regarding mid-morning snack consumption; therefore, we might have only a partial assessment of the number of eating occasions. Nevertheless, there was an afternoon and evening snack question. Among other limitations, loss to follow-up might have introduced selection bias in the longitudinal analysis, and despite adjusting for an array of identified confounders, residual confounding might have biased our results. Moreover, we used biological sex as a proxy for gender as this was the only information available in our cohort and inferred some interpretation regarding gender roles based on the sex differences observed. Finally, although the cohort is population-based, the individuals who answered the 2018 questionnaire, and the 2023 questionnaire were self-selected and display an overall healthier profile than the general baseline GCAT population, being younger and having higher education, which might have incurred selection bias, and impedes generalizability of our results to the population of Catalonia.

## Conclusions

Our results indicated that a later time of first meal and a shorter nighttime fasting duration were associated with a higher BMI, both in cross-sectional and longitudinal analyses. In the cross-sectional analysis, a higher number of eating occasions and later sleep time also showed associations with a higher BMI. We found meal timing patterns that might be related to obesity in women. Given that meal timing patterns are modifiable behaviors, they can be targeted to create chrono-nutritional public health recommendations that would be cost-effective and easy to reach the population. However, the literature is still very new on this topic and more large-scale, prospective and intervention studies are needed.

## Electronic supplementary material

Below is the link to the electronic supplementary material.

Supplementary Material 1

## Data Availability

Data can be provided by contacting the corresponding author. Further details are available from the corresponding author upon request.

## Abbreviations

BMI: Body mass index
GCAT: Genomes for Life
BST: Blood and Tissue Bank
FFQ: Food-frequency questionnaire
MHI5: Mental Health Inventory Score
VIF: Variance inflation factors
GVIF: Generalized variance inflation factors
