# Supplementary material for: Sex-specific chrono-nutritional patterns and association with body weight in a general population in Spain (GCAT study)
Source: Int J Behav Nutr Phys Act. 2024 Sep 12;21:102. doi: 10.1186/s12966-024-01639-x (PMC11396659; doi:10.1186/s12966-024-01639-x)

**Table S1: Baseline and Follow-up population comparison**

|  | Total population | Cross-sectional analysis population | p-value comparing populations |
| --- | --- | --- | --- |
| n | 19392 | 7074 |  |
| Sex (men) | 7916 (40.8) | 2908 (41.1) | 0.69 |
| Age (mean (SD)) | 57.40 (7.31) | 50.80 (7.01) | <0.01 |
| High education (%) | 7244 (37.4) | 3538 (50.0) | <0.01 |

**Table S2: Comparison on mean time of meal-timings and bedtime between weekdays and weekends, GCAT study(N=7074)**

|  | Meal-timings (time of the day) | |
| --- | --- | --- |
|  | Weekday | Weekend |
| Wake-up time (mean (SD)) | 6:57h (0:55) | 8:32h (1:15) |
| Time of first meal (mean (SD)) | 8:29 (1:38) | 9:22h (1:19) |
| Eating midpoint (mean (SD)) | 14:49 (0:57) | 15:24h (0:50) |
| Time of last meal (mean (SD)) | 21:08 (0:45) | 21:26h (0:52) |
| Sleep time (mean (SD)) | 23:30 (0:50) | 00:07h (1:02) |

**Table S3A: Multicollinearity test for model 1^a^**

| Variable | GVIF |
| --- | --- |
| Age | 1.22 |
| Sex | 1.09 |
| rMEDScore | 1.08 |
| Studies | 1.07 |
| Employment | 1.19 |
| Mental Health Score | 1.02 |
| Tobacco consumption | 1.04 |
| Physical activity | 1.01 |
| Time of first meal | 5.47 |
| Nighttime fasting hours | 5.72 |
| Number of eating occasions | 1.18 |

**Table S3B: Multicollinearity test for model 2^b^**

| Variable | GVIF |
| --- | --- |
| Age | 1.23 |
| Sex | 1.09 |
| rMEDScore | 1.09 |
| Studies | 1.08 |
| Employment | 1.21 |
| Mental Health Score | 1.08 |
| Tobacco consumption | 1.04 |
| Physical activity | 1.01 |
| Time of first meal | 6.73 |
| Nighttime fasting hours | 6.79 |
| Number of eating occasions | 1.18 |
| Sleep dissatisfaction | 1.07 |
| Sleep time | 1.27 |

a: Model 1 includes the following variables: age, sex, adherence to Mediterranean diet, education, employment, mental health, smoking habit, physical activity, time of first meal, fasting hours, number of eating occasions, energy intake and baseline BMI.

b: Model 2 includes variables in model 1 + sleep dissatisfaction and bedtime.

GVIF: Generalized variance inflation factors.

**Table S4: Multiple linear regression models assessing the relationship between meal and sleep timing variables and BMI, stratified by time of first meal (GCAT study, cross-sectional analysis)**

|  | Exposures ^a^ | | | | |
| --- | --- | --- | --- | --- | --- |
|  | Time of first meal | Fasting hours | Eating occasions | Sleep dissatisfaction | Sleep time |
| **Overall** |  |  |  |  |  |
| **First meal before 8:30 (n=4326)** | | | | | |
| Model 1 ^b^ | 0.17 [-0.07, 0.40) | -0.15 [-0.33, 0.02] | -0.04 [-0.28, 0.19] |  | |
| Model 2 ^c^ | 0.08 [-0.18, 0.34] | -0.10 [-0.29, 0.09] | -0.03 [-0.27, 0.20] | 0.24 [-0.15, 0.63] | 0.13 [-0.05, 0.31] |
| **First meal after 8:30. (N=2838)** | | | | | |
| Model 1 ^b^ | **0.42 [0.17, 0.66]** | **-0.44 [-0.67, -0.21]** | 0.28 [-0.02, 0.59] |  | |
| Model 2 ^c^ | **0.31 [0.05, 0.58]** | **-0.34 [-0.59, -0.09]** | **0.30 [0.00, 0.61]** | 0.11 [-0.38, 0.59] | 0.20 [-0.01, 0.41] |
| **Men** | | | | | |
| **First meal before 8:30 (N=1710)** | | | | | |
| Model 1 ^b^ | 0.17 [-0.16, 0.50] | -0.16 [-0.41, 0.09] | -0.03 [-0.37, 0.31] |  | |
| Model 2 ^c^ | 0.08 [-0.28, 0.45] | -0.10 [-0.37, 0.17] | -0.01 [-0.35, 0.34] | 0.34 [-0.24, 0.93] | 0.15 [-0.10, 0.41] |
| **First meal after 8:30 (N=1198)** | | | | | |
| Model 1 ^b^ | 0.28 [-0.05, 0.61] | -0.23 [-0.54, 0.07] | -0.02 [-0.42, 0.45] |  | |
| Model 2 ^c^ | 0.26 [-0.09, 0.61] | -0.22 [-0.54, 0.11] | -0.01 [-0.43, 0.44] | 0.43 [-0.29, 1.16] | 0.03 [-0.25, 0.31] |
| **Women** | | | | | |
| **First meal before 8:30 (N=2526)** | | | | | |
| Model 1 ^b^ | 0.15 [-0.18, 0.47] | -0.16 [-0.40, 0.09] | -0.05 [-0.37, 0.27] |  | |
| Model 2 ^c^ | 0.05 [-0.32, 0.43] | -0.11 [-0.37, 0.16] | -0.05 [-0.37, 0.28] | 0.21 [-0.31, 0.72] | 0.13 [-0.13, 0.38] |
| **First meal after 8:30 (N=1640)** | | | | | |
| Model 1 ^b^ | **0.48 [0.13, 0.84]** | **-0.60 [-0.93, -0.27]** | **0.48 [0.07, 0.89]** |  | |
| Model 2 ^c^ | 0.32 [-0.07, 0.71] | **-0.45 [-0.81, -0.09]** | **0.51 [0.10, 0.92]** | -0.03 [-0.68, 0.61] | **0.30 [0.00, 0.61]** |

N: sample size. **Bold: statistically significant.** ^a^ Values are coefficients [95% confidence interval]. Coefficients should be interpreted as difference in BMI for 1 hour increase for each time exposure; for eating occasions it should be interpreted as an increase in 1 eating occasion. Outcome: BMI (kg/m2).

^b^ Model 1 includes the following variables: age, sex, adherence to Mediterranean diet, education, employment, mental health, smoking habit, physical activity, time of first meal, fasting hours, number of eating occasions and energy intake.

^c^ Model 2 includes variables in Model 1 + sleep dissatisfaction and bedtime.

**Table S5: Main characteristics of the participants included in the longitudinal analyses (GCAT study, N = 3,128)**

|  | Overall | Strata: Sex | | | Strata: BMI | | | |
| --- | --- | --- | --- | --- | --- | --- | --- | --- |
|  |  | Women | Men | p | Normal | Overweight | Obese | p |
| **n** | 3128 | 1890 | 1238 |  | 1200 | 1312 | 616 |  |
| **Age (mean (SD))** | 50.85 (6.92) | 50.01 (6.67) | 52.13 (7.11) | <0.001 | 49.49 (6.83) | 51.69 (6.91) | 51.68 (6.72) | <0.001 |
| **Sex (men)** | 1238 (39.6) |  |  |  | 326 (27.2) | 642 (48.9) | 270 (43.8) | <0.001 |
| **Menopause** |  | 1093 (57.8) |  |  | 463 (52.9) | 417 (62.2) | 213 (61.6) | <0.001 |
| **Diet quality: Buckland_rMEDScore (%)** |  |  |  | <0.001 |  |  |  | 0.011 |
| Low adherence | 702 (22.4) | 339 (17.9) | 363 (29.3) |  | 242 (20.2) | 310 (23.6) | 150 (24.4) |  |
| Medium adherence | 1524 (48.7) | 909 (48.1) | 615 (49.7) |  | 576 (48.0) | 634 (48.3) | 314 (51.0) |  |
| High adherence | 902 (28.8) | 642 (34.0) | 260 (21.0) |  | 382 (31.8) | 368 (28.0) | 152 (24.7) |  |
| **Energy intake (kcal/day)** | 2003.30 (580.52) | 1950.96 (562.36) | 2083.21 (598.66) | <0.001 | 2007.05 (574.73) | 1995.19 (567.11) | 2013.30 (619.42) | 0.783 |
| **Education (university)** | 1669 (53.4) | 1017 (53.8) | 652 (52.7) | 0.555 | 715 (59.6) | 649 (49.5) | 305 (49.5) | <0.001 |
| **Employment status** |  |  |  | <0.001 |  |  |  | <0.001 |
| Paid or self-employed | 2451 (79.4) | 1482 (79.4) | 969 (79.6) |  | 995 (83.8) | 1001 (77.7) | 455 (74.8) |  |
| Retired | 234 (7.6) | 98 (5.2) | 136 (11.2) |  | 60 (5.1) | 122 (9.5) | 52 (8.6) |  |
| In charge of the house/family | 100 (3.2) | 96 (5.1) | 4 (0.3) |  | 36 (3.0) | 43 (3.3) | 21 (3.5) |  |
| In a situation of unemployment | 300 (9.7) | 191 (10.2) | 109 (8.9) |  | 97 (8.2) | 123 (9.5) | 80 (13.2) |  |
| **Chronotype** |  |  |  | <0.001 |  |  |  | 0.01 |
| Clearly morning | 807 (26.3) | 454 (24.5) | 353 (29.1) |  | 335 (28.4) | 346 (26.9) | 126 (20.9) |  |
| More morning than evening | 1099 (35.8) | 644 (34.8) | 455 (37.4) |  | 421 (35.7) | 465 (36.2) | 213 (35.3) |  |
| More evening than morning | 854 (27.8) | 537 (29.0) | 317 (26.1) |  | 313 (26.6) | 350 (27.2) | 191 (31.6) |  |
| Clearly evening | 308 (10.0) | 218 (11.8) | 90 (7.4) |  | 109 (9.3) | 125 (9.7) | 74 (12.3) |  |
| **Poor mental health^1^** | 809 (25.9) | 562 (29.7) | 247 (20.0) | <0.001 | 304 (25.3) | 318 (24.2) | 187 (30.4) | 0.014 |
| **Alcohol g (mean (SD))** | 7.92 (10.64) | 5.38 (7.15) | 11.80 (13.54) | <0.001 | 6.72 (8.23) | 8.64 (10.85) | 8.72 (13.72) | <0.001 |
| **Smoking habit (%)** |  |  |  | 0.226 |  |  |  | 0.002 |
| Current | 413 (13.2) | 263 (13.9) | 150 (12.1) |  | 168 (14.0) | 164 (12.5) | 81 (13.1) |  |
| Ex-smoker | 1392 (44.5) | 846 (44.8) | 546 (44.1) |  | 490 (40.8) | 592 (45.1) | 310 (50.3) |  |
| Never | 1323 (42.3) | 781 (41.3) | 542 (43.8) |  | 542 (45.2) | 556 (42.4) | 225 (36.5) |  |
| **Physical activity - mets per week (mean (SD))** | 62.3 [40.8, 94.2] | 62.8 [42.5, 92.0] | 60.8 [38.2, 97.1] | 0.038 | 65.0 [44.1, 95.7] | 63.3 [40.9, 97.0] | 55.2 [32.8, 83.3] | <0.001 |
| **Breakfast consumption (%)** | 3046 (97.4) | 1857 (98.3) | 1189 (96.0) | <0.001 | 1170 (97.5) | 1279 (97.5) | 597 (96.9) | 0.725 |
| **First meal before 8:30h (%)** | 1938 (62.0) | 1176 (62.2) | 762 (61.6) | 0.733 | 777 (64.8) | 795 (60.6) | 366 (59.4) | 0.035 |
| **Time of first meal (median [IQR])** | 8:00 [7:15, 9:15] | 8:00 [7:15, 9:30] | 8:00 [7:20, 9:14] | 0.771 | 8:00 [7:15, 9:15] | 8:00 [7:15, 9:00] | 8:00 [7:25, 9:30] | 0.155 |
| **Time of last meal (mean (SD))** | 21:08 (0:46) | 21:07 (0:45) | 21:10 (0:47) | 0.059 | 21:05 (0:44) | 21:14 (0:47) | 21:09 (0:46) | <0.001 |
| **Fasting hours (median [IQR])** | 11:00 [10:00, 12:00] | 11:00 [10:00, 12:07] | 11:00 [10:00, 12:00] | 0.385 | 11:00 [10:00, 12:00] | 11:00 [10:00, 12:00] | 11:00 [10:00, 12:10] | 0.892 |
| **Hours between wake up and first meal (median [IQR])** | 1:00 [0:30, 2:30] | 1:00 [0:25, 2:15] | 1:00 [0:30, 2:30] | 0.187 | 0:50 [0:25, 2:30] | 1:00 [0:30, 2:20] | 1:00 [0:30, 2:30] | 0.569 |
| **Hours between last meal and sleep (mean (SD))** | 2:17 (0:53) | 2:16 (0:52) | 2:18 (0:53) | 0.252 | 2:16 (0:47) | 2:16 (1:01) | 2:17 (0:53) | 0.75 |
| **Eating midpoint (mean (SD))** | 14:47 (0:56) | 14:46 (0:55) | 14:50 (0:59) | 0.096 | 14:44 (0:56) | 14:52 (0:58) | 14:48 (0:56) | 0.021 |
| **Number of eating occasions (mean (SD))** | 3.42 (0.58) | 3.50 (0.59) | 3.30 (0.56) | <0.001 | 3.41 (0.58) | 3.47 (0.61) | 3.40 (0.58) | 0.069 |
| **Wake-up time (mean (SD))** | 6:57 (0:52) | 6:58 (0:51) | 6:56 (0:55) | 0.499 | 6:55 (0:51) | 6:59 (0:58) | 6:59 (0:50) | 0.155 |
| **Bedtime (mean (SD))** | 23:30 (0:50) | 23:28 (0:50) | 23:35 (0:50) | 0.001 | 23:27 (0:46) | 23:35 (0:58) | 23:32 (0:50) | 0.001 |
| **Sleep dissatisfaction^2^ (%)** | 394 (12.6) | 264 (14.0) | 130 (10.5) | 0.005 | 148 (12.3) | 152 (11.6) | 94 (15.3) | 0.072 |
| **Sleep duration in hours (mean (SD))** | 7:26 (0:52) | 7:29 (0:51) | 7:22 (0:53) | <0.001 | 7:28 (0:49) | 7:24 (0:56) | 7:26 (0:52) | 0.24 |
| **BMI^3^ 2018 (median [IQR]** | 26.13 [23.73, 29.14] | 25.36 [23.05, 28.71] | 26.91 [24.91, 29.54] | <0.001 | 23.10 [21.83, 24.11] | 27.00 [26.01, 28.36] | 32.61 [31.16, 34.96] | <0.001 |
| **BMI^3^ 2023 (mean (SD))** | 25.88 [23.51, 28.80] | 25.31 [22.84, 28.73] | 26.37 [24.35, 28.91] | <0.001 | 23.04 [21.72, 24.24] | 26.63 [25.37, 28.30] | 32.04 [29.73, 34.69] | <0.001 |
| **BMI change (median [IQR]** | -0.26 [-1.34, 0.79] | -0.10 [-1.22, 1.04] | -0.43 [-1.47, 0.40] | <0.001 | 0.02 [-0.75, 0.88] | -0.37 [-1.45, 0.73] | -0.96 [-2.57, 0.72] | <0.001 |

N, sample size; SD, standard deviation; IQR, interquartile range. 1 Poor mental health is defined as a Mental Health Inventory score <60. 2 Sleep dissatisfaction: frequency of participants that answer “no” to the question “are you satisfied with sleep?” in the follow-up questionnaire of the GCAT cohort. 3 BMI: Classified as Normal if BMI < 25, overweight if BMI >=25 & <30, Obese if BMI ≥ 30 kg/m.

**Table S6: Multiple linear regressions models assessing the relationship between meal and sleep timing variables and BMI, stratified by time of first meal (GCAT study, longitudinal analyses)**

|  | Exposures ^a^ | | | | |
| --- | --- | --- | --- | --- | --- |
|  | Time of first meal | Fasting hours | Eating occasions | Sleep dissatisfaction | Sleep time |
| **Overall** | | | | | |
| **First meal before 8:30 (n=1938)** | | | | | |
| Model 1 ^b^ | 0.16 [-0.02, 0.33] | **-0.14 [-0.27, -0.01]** | -0.06 [-0.24, 0.11] |  |  |
| Model 2 ^c^ | 0.09 [-0.11, 0.29] | -0.10 [-0.24, 0.04] | -0.05 [-0.23, 0.12] | 0.18 [-0.12, 0.48] | 0.09 [-0.04, 0.23] |
| **First meal after 8:30. (N=1190)** | | | | | |
| Model 1 ^b^ | 0.12 [-0.07, 0.30] | -0.07 [-0.24, 0.10] | 0.13 [-0.10, 0.36] |  |  |
| Model 2 ^c^ | **0.25 [0.05, 0.45]** | **-0.19 [-0.38, -0.01]** | 0.09 [-0.14, 0.32] | 0.00 [-0.38, 0.38] | **-0.26 [-0.42, -0.09]** |
| **Men** | | | | | |
| **First meal before 8:30 (N=762)** | | | | | |
| Model 1 ^b^ | 0.22 [-0.03, 0.47] | -0.13 [-0.32, 0.06] | -0.19 [-0.46, 0.08] |  |  |
| Model 2 ^c^ | 0.20 [-0.07, 0.48] | -0.12 [-0.32, 0.08] | -0.18 [-0.46, 0.09] | -0.07 [-0.55, 0.40] | 0.02 [-0.17, 0.22] |
| **First meal after 8:30 (N=476)** | | | | | |
| Model 1 ^b^ | **0.25 [0.02, 0.49]** | **-0.23 [-0.44, -0.01]** | -0.13 [-0.45, 0.20] |  |  |
| Model 2 ^c^ | **0.40 [0.15, 0.65]** | **-0.35 [-0.59, -0.12]** | -0.15 [-0.47, 0.17] | **0.74 [0.18, 1.31]** | -0.30 [-0.52, 0.08] |
| **Women** | | | | | |
| **First meal before 8:30 (N=1176)** | | | | | |
| Model 1 ^b^ | 0.12 [-0.12, 0.36] | -0.15 [-0.33, 0.03] | 0.01 [-0.22, 0.24] |  |  |
| Model 2 ^c^ | -0.01 [-0.27, 0.29] | -0.09 [-0.29, 0.10] | 0.02 [-0.21, 0.25] | 0.31 [-0.07, 0.70] | 0.14 [-0.04, 0.32] |
| **First meal after 8:30 (N=714)** | | | | | |
| Model 1 ^b^ | 0.01 [-0.26, 0.28] | 0.04 [-0.20, 0.29] | 0.24 [-0.08, 0.55] |  |  |
| Model 2 ^c^ | 0.15 [-0.15, 0.44] | -0.08 [-0.35, 0.18] | 0.21 [-0.10, 0.53] | -0.31 [-0.81, 0.19] | **-0.26 [-0.49, -0.02]** |

N: sample size. **Bold: statistically significant.**

^a^ Values are coefficients [95% confidence interval]. Coefficients should be interpreted as change in BMI over time for 1 hour increase for each time exposure; for eating occasions it should be interpreted as an increase in 1 eating occasion.

^b^Model 1 includes the following variables: age, sex, adherence to Mediterranean diet, education, employment, mental health, smoking habit, physical activity, time of first meal, fasting hours, number of eating occasions, energy intake and baseline BMI.

^c^Model 2 includes variables in Model 1 + sleep dissatisfaction, bedtime.

**Figure S1: Flow-chart of participants included in the study.**

**
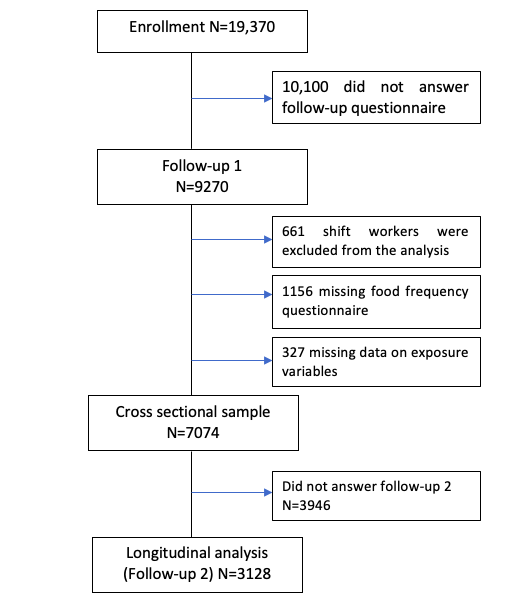
**

**Figure S2A: Correlation between meal and sleep timing variables on weekdays and weekends (GCAT study, N=7074)**

**
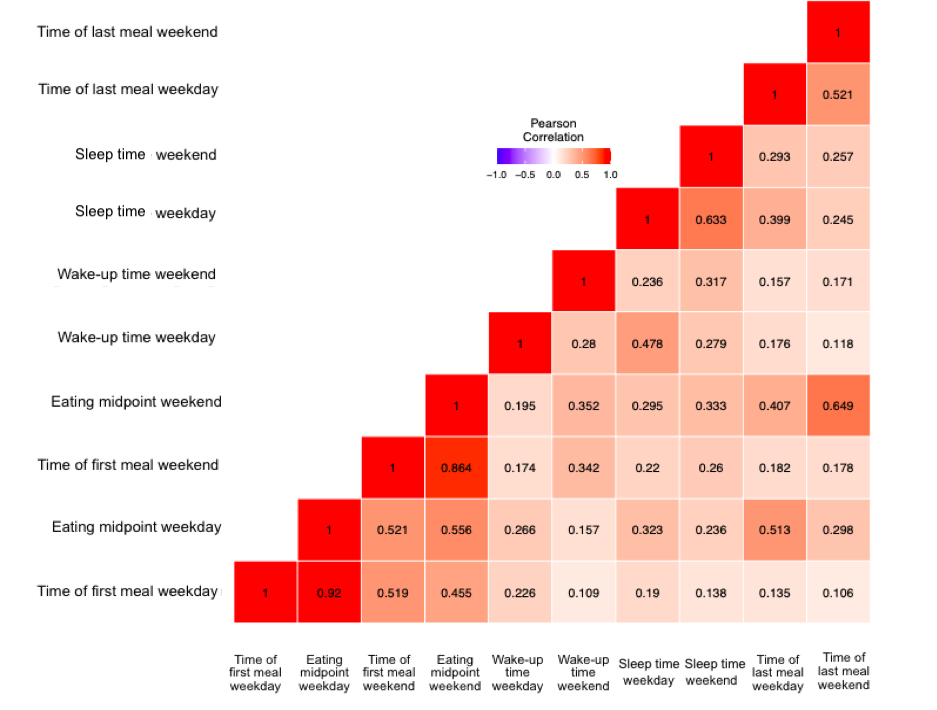
**

**Figure S2B: Correlation between meal and sleep timing variables on weekdays, (GCAT study, N=7074)**

**
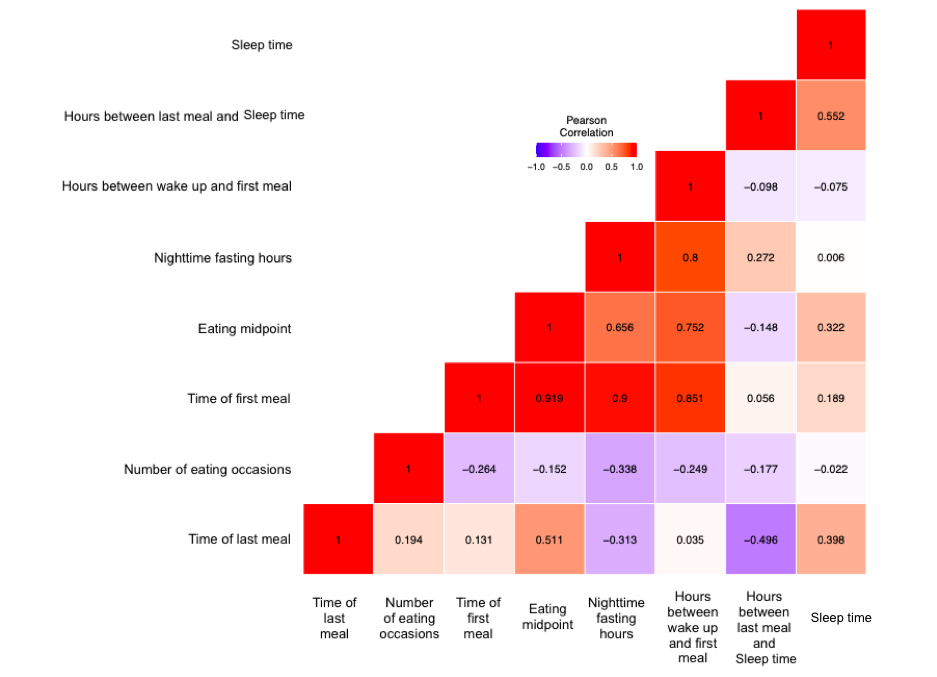
**

**Figure S3: DAG – Chrono-nutritional patterns and their relationship with Body Mass Index**

**
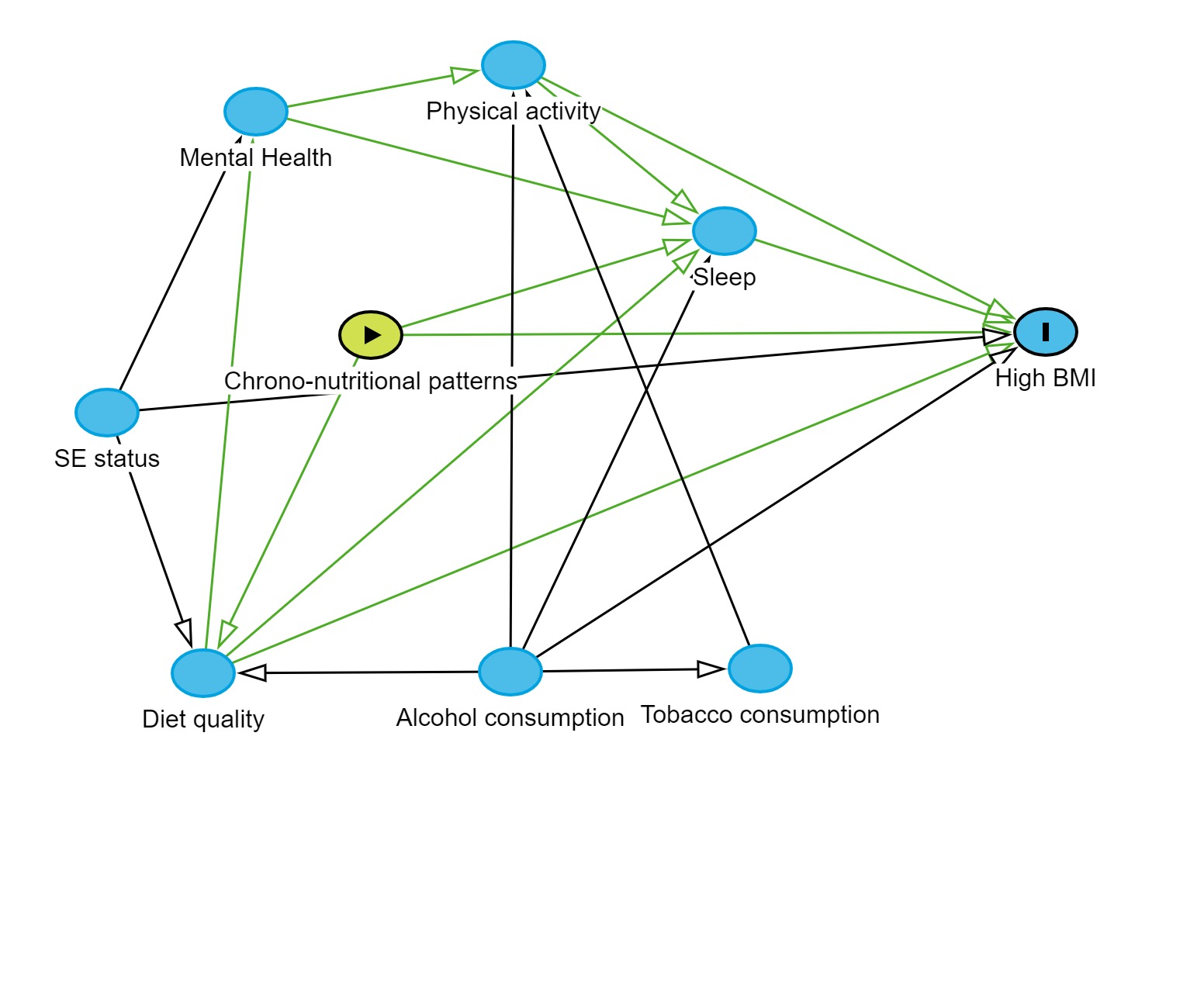
**

**Figure S4A: Silhouette Width for Women**


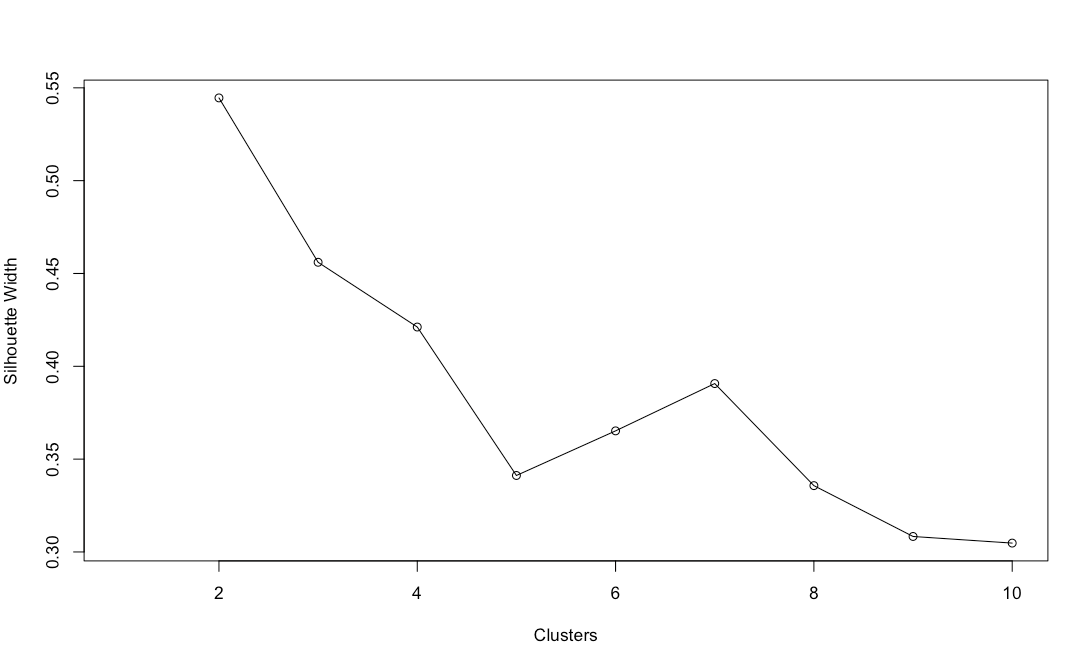


**Figure S4B: Silhouette Width for Men**


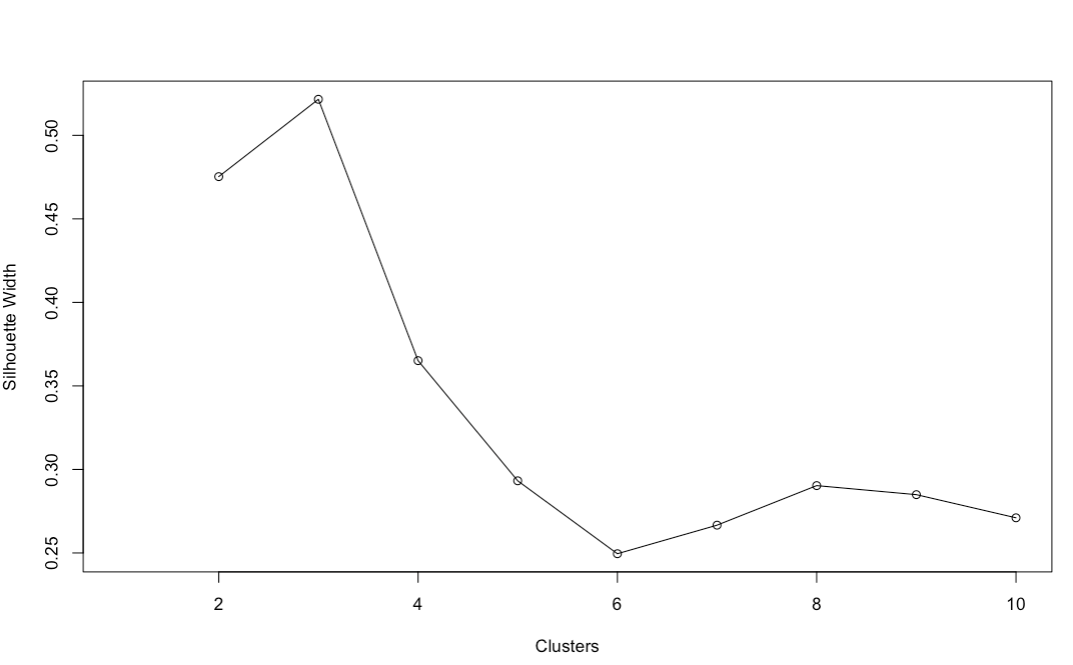

Supplement: Supplementary file 1 — Supplementary Material 1 [file 12966_2024_1639_MOESM1_ESM.docx]
